# A new *Drosophila* model of prolonged inactivity shortens lifespan and impairs muscle function

**DOI:** 10.1038/s41598-025-13446-w

**Published:** 2025-07-31

**Authors:** Jodi Protasiewicz, Sarah Snider, Mousumee Khan, Li Tao, Robert J. Wessells, Alyson Sujkowski

**Affiliations:** 1 Department of Pharmacology, Wayne State University School of Medicine, Detroit, MI 48201 USA; 2 Department of Physiology, Wayne State University School of Medicine, Detroit, MI 48201 USA; 3 Department of Ophthalmology, Visual, and Anatomical Sciences, Wayne State University School of Medicine, Detroit, MI 48201 USA; 4 Department of Physical Therapy, College of Pharmacy and Health Sciences, Wayne State University Eugene Applebaum, Detroit, MI 48201 USA

**Keywords:** Prolonged inactivity, Confinement, Exercise, *Drosophila melanogaster*, Physiology, Ageing, Genetics

## Abstract

Prolonged inactivity due to medical conditions can cause chronic muscle disuse and lead to physical incapacity and poor quality of life. Here, we developed a *Drosophila* model of confinement inactivity (CI) to observe its effects on lifespan and muscle function. We found that, similar to mammalian models and humans, CI negatively impacted longevity and function in *Drosophila.* Confined flies had impaired mobility, shorter lifespan, and reduced muscle integrity compared to their freely mobile siblings. These findings establish a new, highly efficient platform for studying long term effects of chronic sedentary behavior and muscle disuse in the genetically tractable *Drosophila* model. In addition, we found that temporarily removing flies from CI for scheduled bouts of forced physical exercise ameliorated negative effects, in part by improving muscle homeostasis. Finally, we tested whether muscle overexpression of 3 exercise-responsive genes, *dPGC-1α*, *dFNDC5*, or *dSesn*, could prevent the negative impact of CI on fly aging, even without physical exercise. We previously established that overexpression of these factors phenocopies exercise effects in aging wild-type and disease model flies. We found that when overexpressed in muscle, *dSesn* prevented premature declines in endurance, and *dFNDC5* protected speed and endurance. This new model can be used in the future for mechanistic studies to identify preventative and therapeutic targets for diseases associated with chronic inactivity.

**Supplementary Information:**

The online version contains supplementary material available at 10.1038/s41598-025-13446-w.

## Introduction

 Sedentary lifestyle has become one of the major risk factors for premature death across the world^1^. For many individuals, this issue can be well-addressed with public-health initiatives to motivate more people to boost their daily activity levels. However, a substantial subset of individuals suffers from prolonged periods of inactivity that are not under their control, including those with mitochondrial diseases^2^neurodegenerative diseases^3^accidents or injuries with prolonged recovery periods^4^or other causes^5^. Patients under temporary bedrest can be treated with exercise after improvement of their condition to gradually restore healthy muscle function and metabolism^6,7^. Unfortunately, for patients with long-term conditions that do not have definitive cures, physical therapies may be inaccessible^8^ or even worsen their condition^9–11^. For those patients, secondary metabolic and functional impairment due to inactivity can lead to deterioration of their overall health condition and quality of life additively with the original illness or injury. Therefore, novel therapeutic options to ameliorate the effects of prolonged inactivity in these patients are highly desirable.

Prolonged sedentary periods can cause adverse effects on skeletal muscle, both structurally^12^ and functionally^13^. Muscle mass is lost when the balance between protein synthesis and degradation is disrupted^14^. During acute inactivity, protein synthesis remains relatively stable and muscle loss occurs via the ubiquitin proteasomal system^15^. In contrast, reduced protein synthesis becomes the primary driver of muscle atrophy after prolonged periods of inactivity^14^. Muscle function is known to suffer more profoundly than can be explained by mass reduction alone, indicating significant metabolic and structural deficits^16^. Substantial work has been done in both rodent models^17,18^ and humans^19,20^ to explore the efficacy of potential therapeutic interventions that address the needs of patients with prolonged physical inactivity. However, rodent studies often engage only one common lab genotype, meaning the conclusions may not be generally applicable, whereas human studies suffer from the highly divergent genetic backgrounds and life histories of patients that are difficult to control for.

*Drosophila melanogaster* can be a useful model in such cases because of the available genetic techniques and ability to expand studies across several divergent genotypes in large numbers. Here, we describe a model of confinement inactivity (CI), utilizing *D. melanogaster* to characterize the effects of prolonged activity reduction. This new model of sedentary behavior leverages the well-established benefits of *Drosophila*: short lifespan, straightforward genetics with high human disease relevance, and well characterized, easily measured phenotypic traits. Perhaps more importantly, *Drosophila* allow us to identify conserved mechanistic pathways to prevent and treat chronic diseases arising from inactive lifestyles, more cheaply and efficiently than is possible in mammalian models and humans. We find that CI reduces endurance, climbing speed and longevity with corresponding changes in muscle structure. On the other hand, flies that are restrained for most of their life, but briefly released five times a week to undergo an exercise program, live longer and retain endurance, climbing speed, and muscle actin integrity better than age-matched, unexercised and confined siblings. These improvements occur via improved muscle homeostasis mediated in part by partial rescue of reduced AKT activity in CI flies. Lastly, to support the model’s utility in identifying cellular programs that may prevent inactivity-related declines, we overexpress three exercise-response genes that we previously established can substitute for exercise in wild-type and disease model flies: the key regulator of mitochondrial biogenesis PGC-1⍺ (*spargel*,* srl*)^21,22^the mTOR modulator Sestrin (*dSesn*)^23^and the metabolic regulator FNDC5 (*Idit*)^24^. This accessible *Drosophila* model of CI opens the door for investigations in widely ranging fields that may identify mechanistic targets that can be leveraged toward prevention and treatment of disease in chronically inactive patients.

## Results

**Fig. 1:**
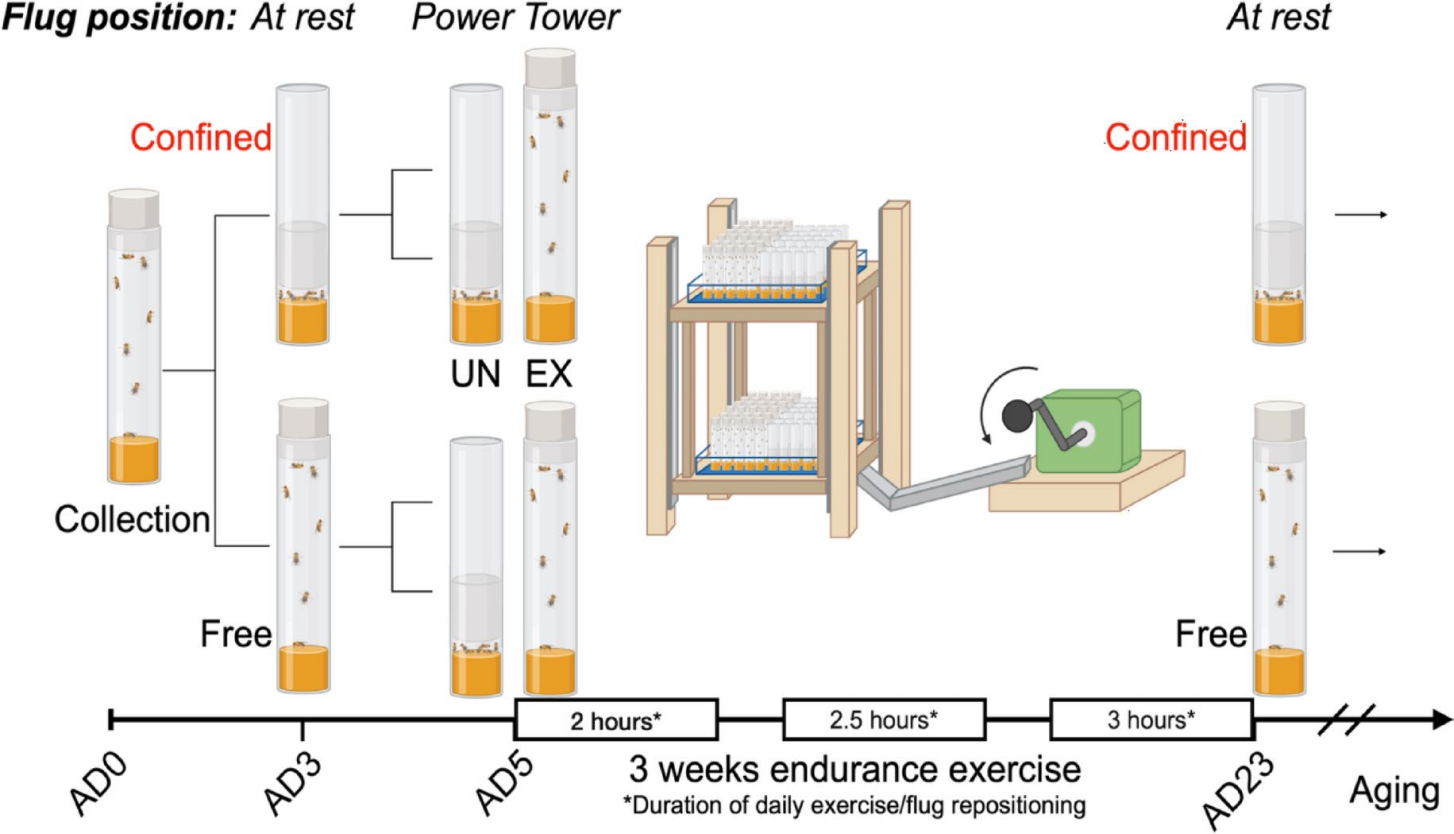
Illustration of confinement inactivity in *Drosophila****.*** Age matched flies are collected on eclosion and then separated into “Confined” and “Free” cohorts. Confined flies are housed with a soft plug positioned low in the vial to restrict movement. Free flies can move about the length of the vial. Vials are then further separated into exercised and unexercised cohorts. Starting on adult day 5, flies are removed from the incubator and plugs are repositioned based on whether Confined or Free cohorts are exercised (plug up) or unexercised (plug down). After daily completion of the exercise program plugs are returned to their starting positions (plug down: Confined, plug up: Free) and returned to the incubator.

### **Confinement inactivity in*Drosophila*negatively impacts longevity and function**

We modeled prolonged inactivity in *Drosophila* with a confinement protocol where groups of flies are restricted to a 5 mm x 25 mm (~ 2,500 mm^3^) space by placing a foam stopper low in normal housing vials. Control flies are free to move throughout a 95 mm x 25 mm (~ 45,000 mm^3^) space. Confined flies can easily get food and execute minimal movements but cannot freely climb or perform any kind of regular exercise. This chronic confinement is much more extreme than the protocol for “unexercised flies”^25^ in which flies are restrained only during the hours that their siblings are on the exercise machine, then allowed to resume normal laboratory behavior. The intention is to model a situation in which a patient is chronically sedentary, where they can perform some movements but experience no regular exercise. The confinement inactivity (CI) protocol is illustrated in Fig. 1.

**Fig. 2:**
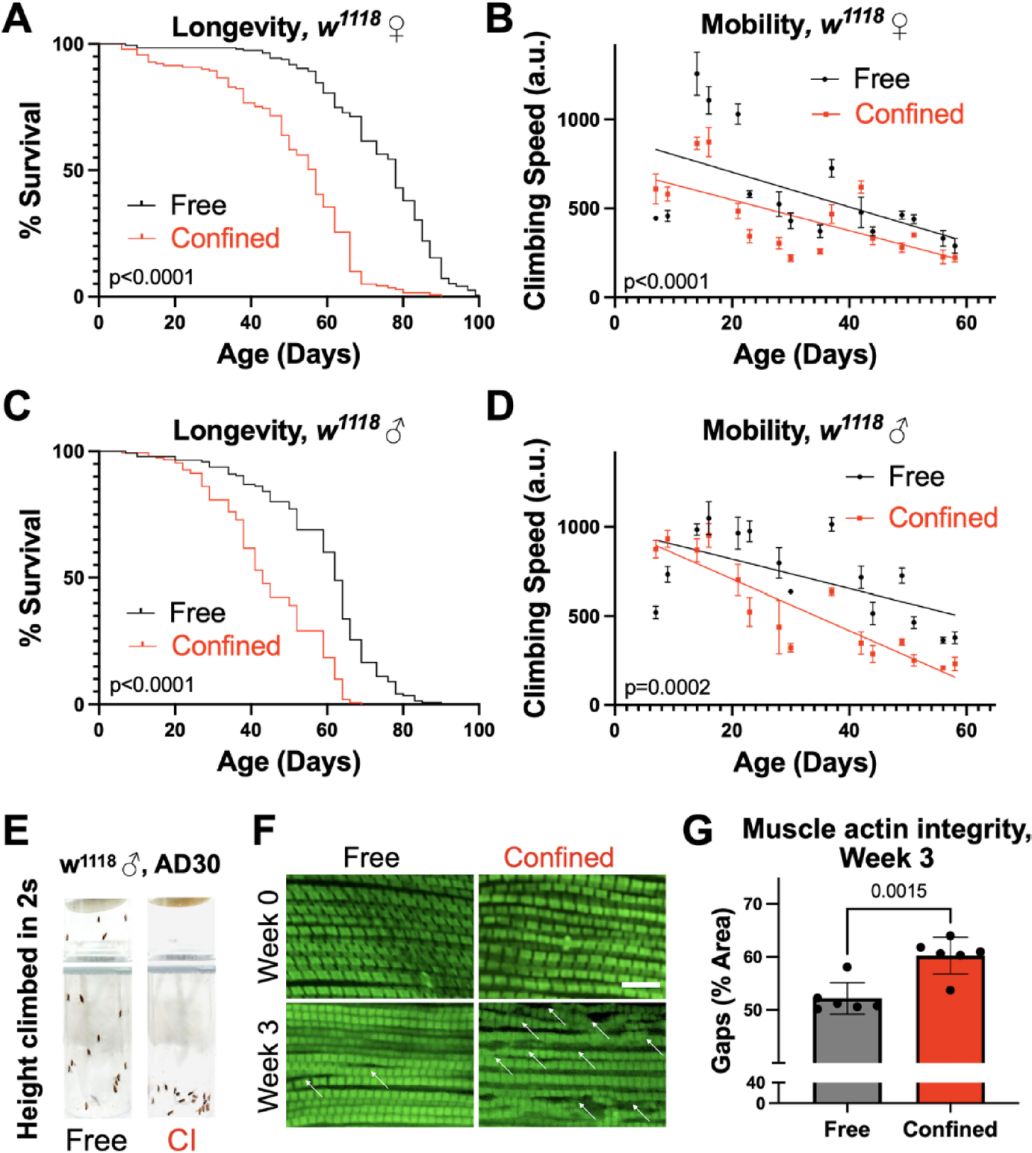
Confinement induced inactivity negatively impacts *Drosophila* health. Confined flies have reduced lifespan **(A**,** C)** and mobility **(B**,** D)** compared to age-matched, freely mobile siblings. **(E)** Representative photos of climbing speed in 2 s in 30-day old Free and Confined (CI) flies. Longevity analyzed by log-rank, mobility analyzed by linear regression, looking for differences in slope and intercept. *n* > 200 flies per experiment; mobility and longevity experiments were repeated a minimum of 3 times. **(F)** Representative 100x images of phalloidin stained indirect flight muscle at weeks 0 (upper panels) and 3 (lower panels). White arrows point to gaps in actin filaments, quantified in **(G)**. *n* > 5 for each of at least 3 biological replicates. Error bars represent SEM, p-value from student T-test. Scale bar = 10 μm.

First, to characterize the systemic effects of chronic sedentary conditions in flies, we measured longevity and climbing speed in a common laboratory strain (*w*^*1118*^) of female and male flies (Fig. 2). Confinement inactivity markedly shortened lifespan in females (Fig. 2A) and males (Fig. 2C). Similarly, both female (Fig. 2B) and male flies (Fig. 2D, E) under CI had worse mobility than age-matched, freely mobile siblings, with males experiencing a more rapid rate in age-related decline (linear regression for slope, *p* = 0.0002). These changes are consistent with observed effects of prolonged inactivity in vertebrates^12,26^.

Next, we looked at muscle structure in flies under CI since prolonged skeletal muscle inactivity is known to result in significant muscle atrophy^27^. Phalloidin staining of indirect flight muscle (IFM) revealed disorganized actin fibers, with “gaps” forming in CI flies by 3 weeks of age (Fig. 2F, bottom rows, gaps indicated by arrows, quantified in 2G).

### Exercise improves lifespan and mobility in chronically inactive flies

**Fig. 3:**
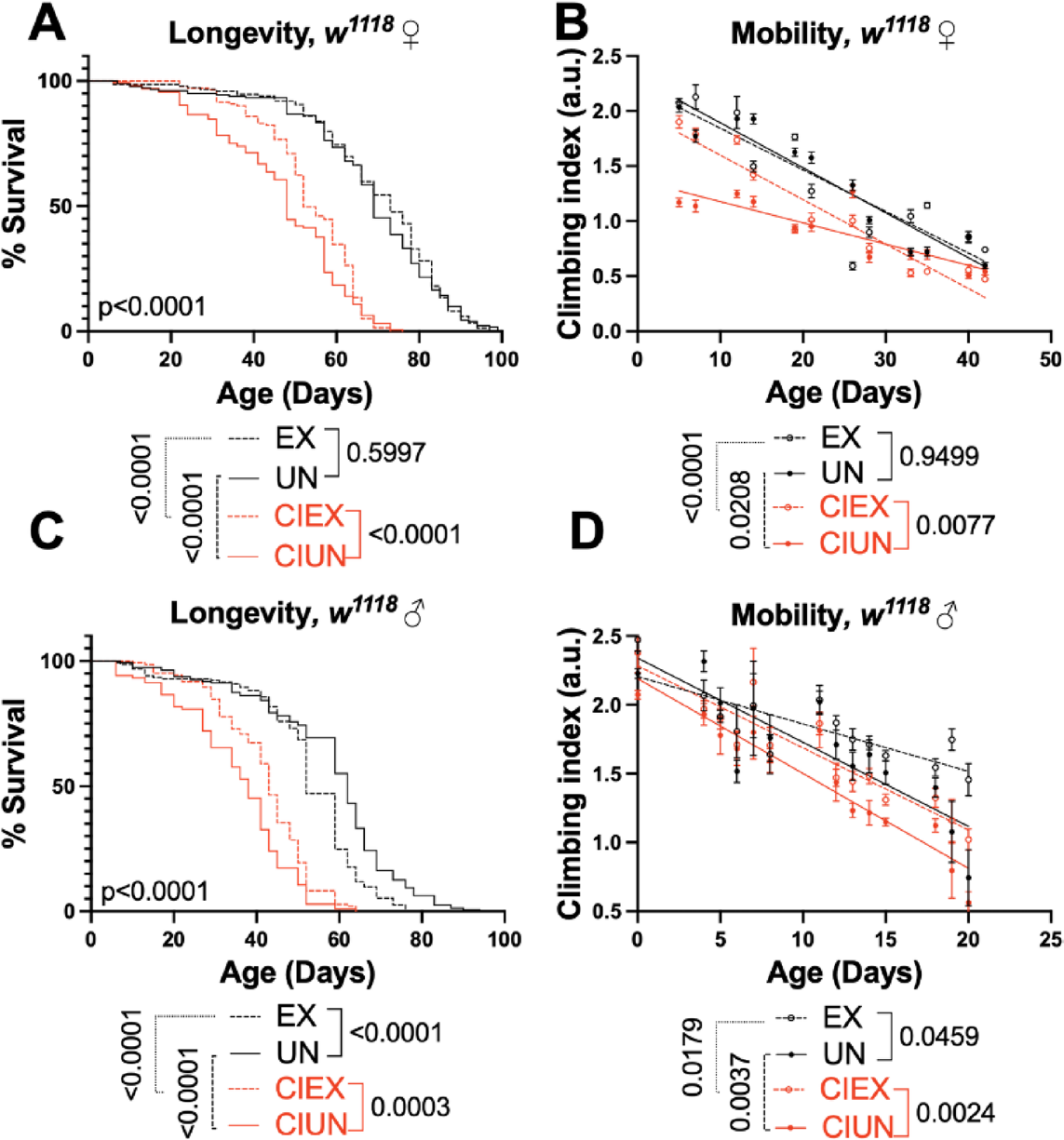
Exercise improves health in mobility restricted flies**. (A)** Lifespan is improved in CI female flies that complete 3 weeks of exercise when compared to unexercised, restrained siblings. **(B)** In this biological cohort, exercised female flies that undergo CI improve mobility but do not reach the capacity of freely moving control flies. Exercised male flies that undergo CI have better lifespan **(C)** and mobility **(D)** than unexercised siblings. *n* > 200 flies per experiment, exercise, mobility and longevity experiments were repeated a minimum of 3 times. Longevity analyzed by log-rank, mobility analyzed by linear regression, looking for differences in slope and intercept.

Breaking up prolonged sedentary periods with exercise improves physical performance in humans^28,29^so we examined whether periodic exercise could ameliorate the negative impact of prolonged inactivity in confined flies. Once again, CI shortened lifespan and negatively affected mobility in both females (Fig. 3A, B, S1 A, B) and males (Fig. 3C, D, S1 C, D) but exercise significantly improved both parameters. Interestingly, exercise partially extended male and female longevity in confined groups, but in male flies, exercise completely restored climbing speed to the level of unexercised, freely mobile siblings. We previously established that our exercise program also improves age-related declines in endurance^22,30^and that exercised male and female flies have sexually dimorphic adaptations to mobility (endurance and climbing speed^31^), a phenomenon we also observed here in our freely mobile exercised control females (Fig. 3B, mobility in exercised female CI flies did not improve in a second repetition, see Fig. S1B). Because both humans and flies have phenotypic variability that depend on genetic background, we decided to repeat our exercise experiments in males from 2 additional genetic backgrounds, *y*^1^*w*^1^ and *Canton S*. Exercised male flies of diverse genotypes have robust and repeatable improvements in endurance^22,23,31–37^making them a good model for examining exercise effects on CI.

**Fig. 4:**
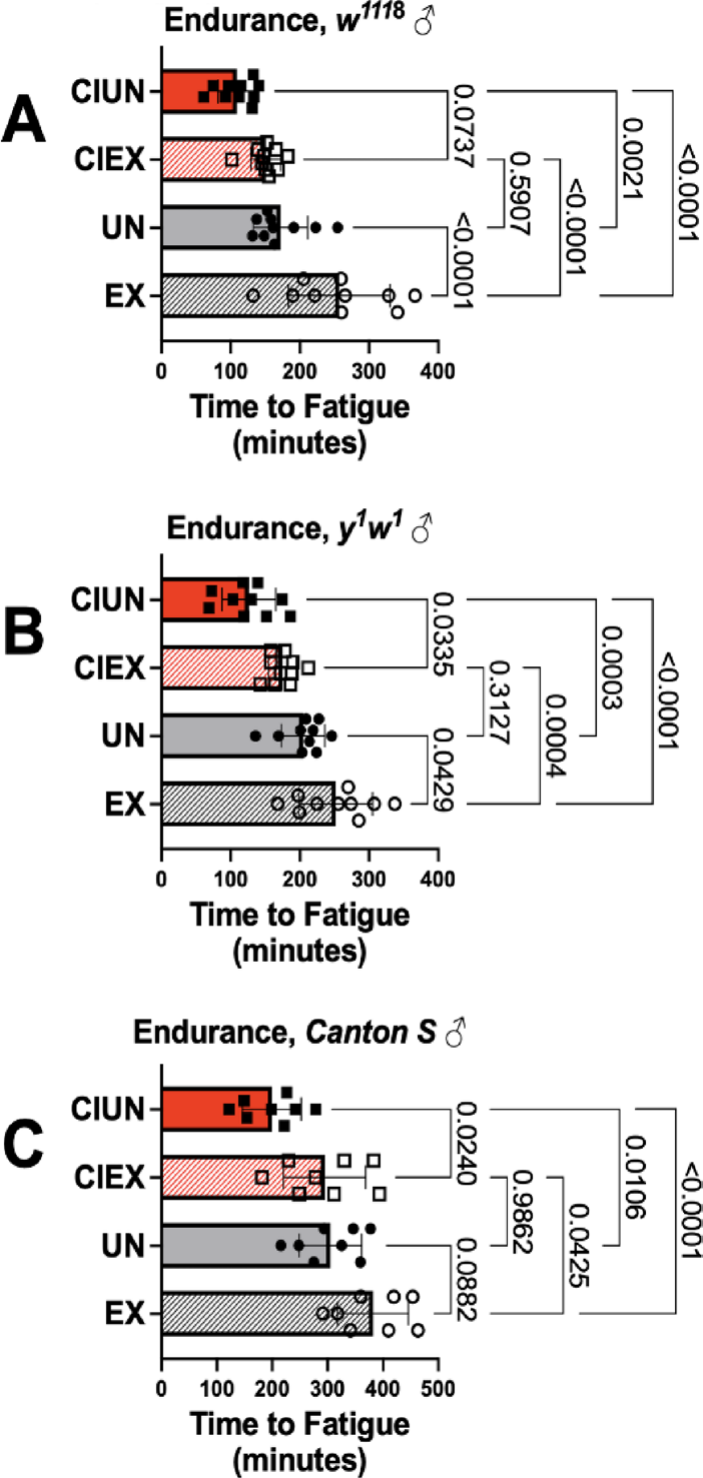
Exercise partially rescues endurance in confined flies**. (A)** Restrained, unexercised *w*^*1118*^ flies have lower endurance than unexercised, freely moving control flies (compare solid red bars to solid grey bars), but exercise training improves it (compare hatched red bars to solid grey bars). Endurance benefit does not equal that of exercised, freely moving flies, however (compare red hatched bars to grey hatched bars). Similar results were seen in **(B)** *y*^1^*w*^1^ and **(C)** *Canton S* flies. Analyzed by ANOVA with Tukey post-hoc test. Main effect *p* < 0.0001 for all groups.

Endurance in unexercised CI flies was lower than in age matched, freely mobile unexercised siblings in all 3 genotypes, *w*^*1118*^ (Fig. 4A), *y*^1^*w*^1^ (Fig. 4B) and *Canton S* (Fig. 4C, compare solid grey bars to solid red bars). Periodic endurance exercise improved endurance in exercised CI flies (CIEX) across genotypes, although improvement did not equal that of exercised, freely mobile flies. Similarly, shortened lifespan in CI flies was partially improved by exercise in all groups (Supplemental Fig. S2).

### Exercise prevents atrophy in chronically inactive flies

**Fig. 5:**
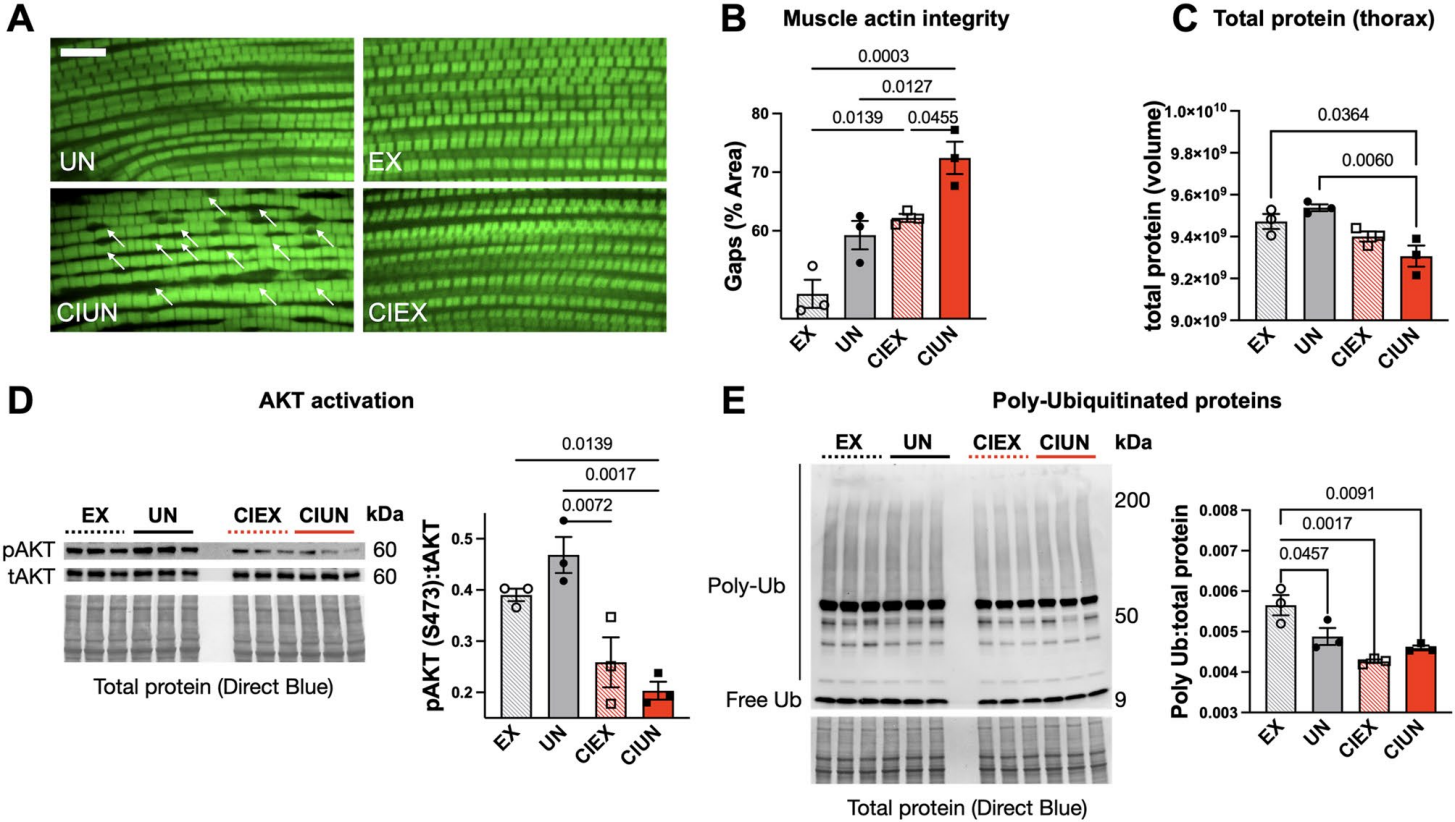
Exercise preserves muscle structure in confined flies.** (A)** Three weeks of exercise in restrained flies significantly improves muscle actin integrity (quantified in **(B**, ANOVA, *p* = 0.0006**)**). Representative 100x images. Gaps in actin filament structure indicated by white arrows. Scale bar = 10 μm. (**C)** Total protein from isolated thorax muscle is lower in unexercised CI flies compared than freely mobile controls or exercised CI siblings. 10 thoraxes per biological replicate, 4 biological replicates performed, ANOVA, *p* = 0.0074. **(D)** Western blot of pAKT: AKT indicates lower ratios in CI flies than in controls, but CIEX flies are statistically similar to exercised, freely mobile siblings. 10 thoraxes per biological replicate, 3 biological replicates and 2 technical replicates performed ANOVA, *p* = 0.0014 **(E)** Western blot of Ubiquitinated proteins show lower levels of poly-Ub in CI flies. 10 thoraxes per biological replicate, 3 biological replicates and 2 technical replicates performed, ANOVA, *p* = 0.0022. Error bars depict SEM. Histograms analyzed by ANOVA with Tukey post hoc comparison.

We next examined muscle structure to see if exercise could protect against degeneration during confinement. After 3 weeks of exercise, phalloidin-stained IFM in freely mobile exercised and unexercised flies looked similar (Fig. 5A). In contrast, unexercised CI flies had more disorganized muscle fibers and gaps that were not observed in exercised CI flies (Fig. 5A bottom panels, quantified in Fig. 5B). Furthermore, total protein content from isolated thoraxes (*Drosophila* thorax is heavily enriched for muscle^38)^ was significantly lower in unexercised CI flies than exercised siblings or freely mobile control flies (Fig. 5C). Taken together, these data support a protective effect of exercise against CI-induced atrophy in *Drosophila*.

In order to determine mechanistically how exercise prevents muscle loss, we quantified the ratio of phospho-AKT (pAKT) to total AKT (tAKT) and Ubiquitinated proteins (Ub) in isolated fly thoraxes. Activated AKT (higher pAKT; tAKT ratio) is associated with muscle protein sythesis, while increased activity of the Ub-proteosomal system is associated with muscle protein degredation^14^. Both exercised and unexercised CI flies have significantly lower pAKT: AKT than age-matched, freely mobile control siblings, an effect that was partially rescued in exercised CI flies (Fig. 5D, source data in Fig. S3A). Poly-Ub was lower in CI flies whether exercised or not, suggesting dysfunction of the Ub-proteosomal pathway that is not improved with physical exercise (Fig. 5E, source data in Figure S3B). Our results indicate that 2–3 h of exercise a day provides substantial protection against muscle degeneration by improving deficient protein synthesis and restoring muscle homeostasis, even when flies are restrained the rest of the day.

### **Exercise responsive genes preserve mobility in unexercised**,** chronically inactive flies**

**Fig. 6:**
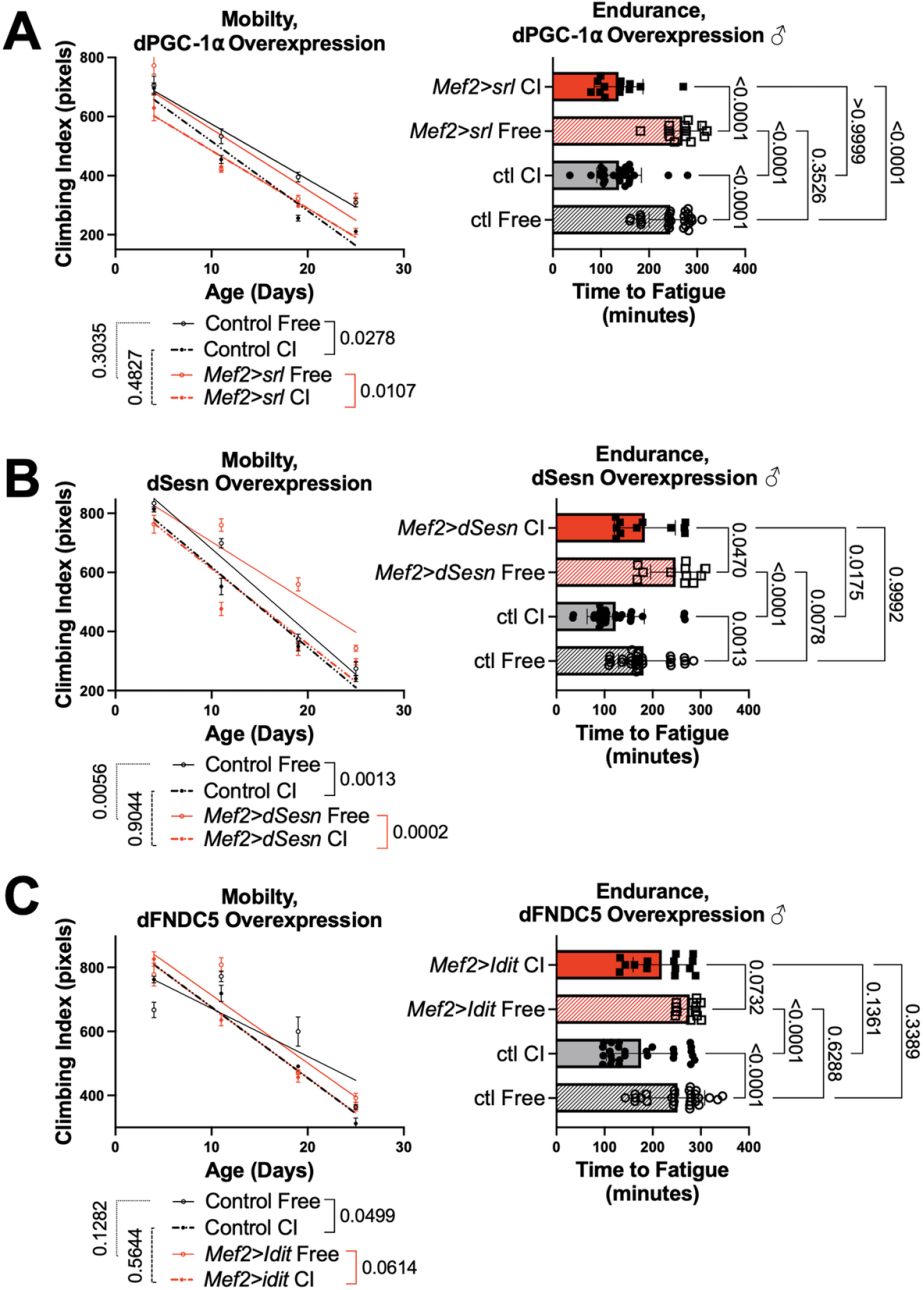
Muscle specific expression of *dSesn* or *Idit* improve mobility in confined flies.** (A)** Flies overexpressing *dPGC-1α* in muscles (*Mef2 > srl*) have similar climbing speed and endurance to background control flies whether or not they are chronically confined. **(B)** Flies overexpressing *dSesn* in muscles (*Mef2 > dSesn* Free) have better mobility than age-matched, freely mobile background control flies. *Mef2 > dSesn* CI flies have similar mobility to Control CI siblings. *Mef2 > dSesn* CI flies have similar endurance to freely mobile background controls (ctl Free). **(C)** Flies overexpressing *dFNDC5* in muscles (*Mef2 > Idit*) have similar speed and endurance to freely mobile background controls whether or not they are confined. *N* > 200 flies for all experiments. Mobility analyzed by linear regression, significance reported for differences in intercept or slope. Mean endurance is analyzed by ANOVA with Tukey post-hoc comparison. Main effect, *p* < 0.0001 all groups, error bars depict SEM.

 We previously established that the conserved exercise response genes *dPGC-1α* (*spargel*,* srl*)^39^*Sestrin* (*dSesn*)^23^and *FNDC5* (*Iditarod*,* Idit*)^24^ are induced in exercising fly muscle and can substitute for exercise to improve mobility in unexercised flies^23,24,39^. Here, we investigated whether muscle-specific overexpression of *srl*,* dSesn*,* or Idit* (Supplemental Figure S4) was sufficient to protect physical performance during CI. Confined flies with muscle-specific *dPGC-1α* expression (*Mef2 > srl*) had similar climbing speed (Fig. 6A, left panels) and endurance (right panels) to age matched CI background controls. Freely mobile *Mef2 > dSesn* flies had higher baseline climbing speed and endurance than background control flies as previously reported^21,23^but muscle specific *dSesn* expression did not improve climbing speed when flies were confined (Fig. 6B, left panels). On the other hand, endurance in confined *Mef2 > dSesn* flies was partially rescued and similar to freely mobile background control flies, although it did not reach the endurance of freely mobile flies with muscle specific *dSesn* overexpression. Lastly, we tested climbing speed and endurance in flies with muscle specific *dFNDC5* overexpression (*Mef2 > Idit*, Fig. 6C). Confined background control flies once again had decreased climbing speed and endurance compared to age-matched, freely mobile siblings. Interestingly, *dFNDC5* overexpression in muscles was able to prevent CI-induced impairments in both speed and endurance, with confined *Mef2 > Idit* flies performing as well as freely mobile background controls.

## Discussion

Prolonged inactivity is a major health problem contributing to a variety of human diseases^2,3^. Public health initiatives aimed at promoting even short exercise bouts to break up sedentary behavior can be effective in combating inactivity. However, such initiatives are not applicable to those who have extenuating circumstances that force them into prolonged or chronic physical inactivity and muscle disuse. Individuals that are disadvantaged by factors outside of their own control such as the elderly, those suffering from neuromuscular or metabolic disease, hospital patients bedridden with severe injury or illness, or even astronauts exposed to prolonged periods of microgravity commonly suffer from muscular atrophy as a by-product. Protection against chronic inactivity is therefore of the utmost importance to improve quality of life and reduce mortality in a variety of populations, yet the mechanisms protecting against chronic muscle disuse are not fully understood.

*Drosophila melanogaster* has emerged as an attractive model organism to study how exercise can improve health and longevity^11,21–23,31,32,35–37,40–42^but until now there existed no model of chronic inactivity in adult flies. This new model will provide a platform for elucidating the mechanistic and molecular effects of chronic inactivity and can provide a useful foundation upon which to test a variety of potential therapeutic interventions. Here, we show that confining adult flies to a small living space markedly reduces both longevity and function, and that exercise is able to combat the negative impact of chronic inactivity.

It is perhaps surprising that while confined flies have phenotypes that parallel muscle degeneration in higher organisms, ubiquitination is not elevated in our chronically inactive flies. Aging fly muscle progressively accumulates insoluble poly-ubiquitinated proteins^43^ and muscle fibers degenerate over time via proteolysis^43^ and necrosis^44^changes that correlate with functional decline^43,44^. Muscle atrophy occurs in humans when the balance between muscle synthesis and breakdown is disrupted, and in early stages of bedrest muscle loss is primarily driven by a shift toward muscle breakdown^14,15^. However, during chronic inactivity lasting for several weeks or more, the relationship between the ubiquitin-proteosomal system and muscle atrophy is more complex. Our study in *Drosophila* more closely parallels long-term muscle disuse, as flies are confined from the time they eclose all the way until their death, only being released for short bouts of exercise. Nevertheless, we see marked improvements in health and longevity of confined flies when they are allowed short exercise breaks, despite exercise only lasting for 3 weeks of the lifelong confinement protocol. Our results highlight the potent therapeutic impact of exercise and open the door for more in-depth mechanistic studies.

We used here a model of forced exercise and a representative set of assays selected for their defining character as universally expected changes associated with enforced sedentary periods. We reasoned that if the model is legitimately mimicking the effects of controlled inactivity, then it should show changes to actin structure and protein turnover, and these should be accompanied by reduced muscle function in whole-animal physiology. Some limitations of the experimental system are that it is not well-suited to address impacts of voluntary exercise, and the exact number of “steps” taken by each individual tend to be different, necessitating use of large numbers to get reproducible averages. Other ways of inducing forced exercise besides the Power Tower include the TreadWheel^45^which uses rotation of vials to induce negative geotaxis, and methods that roll the vial to stimulate movement. Activity can also be tracked by using beam-tracking to estimate movement parameters, or can be quantitated at the individual level through several varieties of movement-tracking software^46–48^. Given the abundant biological material and varied techniques available in *Drosophila*, we anticipate our confinement model to be used in the future toward a variety of systematic and mechanistic investigations with high therapeutic relevance.

Previous research has started to explore the role of Sestrin^49,50^ *dPGC-1α*^51^ and FNDC5^52^ during prolonged inactivity, but much remains to be discovered. Here, we found that while *dPGC-1α* (*srl*) did not prevent the negative effects of chronic inactivity, both *Sestrin* (*dSesn)* and *FNDC5* (*Idit*) in muscle provide some benefit. It was recently discovered that spontaneous muscle fiber degeneration in aging flies is characterised by mitochondrial swelling, a morphological hallmark of necrosis^44^. In our study, muscle overexpression of *Idit*, a significant regulator of mitochondrial homeostasis^24,53–55^conferred the most complete protection against confinement induced mobility loss.

We previously established that *dSestrin*^21,23^*spargel*^39^ and *Iditarod*^24^ are necessary and sufficient for mediating beneficial effects of chronic exercise in unconfined flies^21,23^and these effects are also conserved in mice^21,23,54,56,57^. *Sestrin* overexpression in muscle prevented muscle disuse and weakness in mice through the upregulation of autophagy and AKT activation^49^. In *Drosophila*, *dSesn* overexpression in muscle increased running endurance and performance through the upregulation of AKT phosphorylation^23^. Here, AKT activation contributed to the protective effect of exercise in chronically inactive wild-type flies, but *dSesn* overexpression improved endurance without any benefit to climbing speed. Previous work showed that AKT activation by Sestrin prevented ubiquitination dependent muscle loss^49^but here we found poly-Ub levels were low in confined flies whether exercised or not. It is possible that in our sustained confinement protocol, protection against muscle loss critically depends on activating protein synthesis, and that Sestrin activation inhibits proteosomal breakdown too much to provide maximum benefit. Further work will be needed to address the exact mechanistic reasons for the efficacy of rescue.

## Methods

### Fly Stocks and maintenance

Wild-type *w*^*1118*^ (Stock #3605), *y*^1^*w*^1^ (Stock #1495), and *Mef2 Gal4* (Stock #27390) were obtained from the Bloomington Drosophila Stock Center (Bloomington, IN, USA). *Canton* S was gifted from Rolf Bodmer (Sanford Burnham Prebys), *UAS-srl* was a gift from Dr. David Walker (University of California, Los Angeles, CA, USA) and *UAS-dSesn* and *UAS-Idit* from Dr. Jun Hee Lee (University of Michigan, Ann Arbor, MI, USA).

The flies were maintained on 10% sugar-yeast medium at 25 °C under controlled 50% humidity and 12-hour light/dark cycle. Wild type flies (*w*^*1118*^, *y*^1^*w*^1^ *and Canton* S) were age matched by collecting within 3 h of eclosion and thus reproductively immature. Experimental flies for the muscle specific overexpression experiments were F1 flies heterozygous for the *Mef2 Gal4* enhancer and upstream activating sequence (UAS) from one of the lines described above, also collected within 3 h of eclosion. Control flies for all Gal4-UAS experiments consisted of the Gal4 or UAS lines crossed into the appropriate background control, *Attp*, *y*^1^*w*^1^or *w*^*1118*^ using the same age-matching technique as in wild-type flies.

### Confinement protocol

The confinement protocol can be visualized in Fig. 1. Prior to confinement, a total of 1200 flies per genotype were collected under light CO_2_ anesthesia within 3 h of eclosion and separated into vials containing 20 flies which were later divided into two groups: Free (plug up) or CI (plug down), with 600 flies per cohort. Flies enter this protocol 5 days after eclosion to allow time for adult development to take place normally. For CI cohorts, foam plugs were pushed down to near the bottom of the vial so that flies could easily access food and retain minimal movements but had no room to jump, fly or execute any kind of regular exercise. On the other hand, free flies could freely climb inside the vial as the foam plugs were kept at the top of the vial. Confinement experiments were carried out for the entire lifespan of the flies and all groups were housed in a 25 °C incubator except during exercise training.

### Exercise training

Exercise training was performed for three weeks as previously described^25^. Briefly, at least 600 flies were anesthetized using CO_2_ within 3 h of eclosion and immediately transferred into vials of 20 flies/vial which were separated into two groups of at least 300 flies: control exercised (EX) and control unexercised (UN) groups. CI flies were also separated into two groups of at least 300 flies: Confinement inactivity with exercise (CIEX) and Confinement inactivity without exercise (CIUN) groups. Regardless of confinement grouping, exercised and unexercised groups were both placed on the training device 5 times per week, with the time of exercise ramping up each week. The stopper was placed low in the vial for the unexercised groups and high in the vial for exercised groups, as previously described^11,21,23,31–34,36,37,58–61^. The UN group had the plugs pushed down during the training bouts only, whereas the CIUN group had the foam plugs pushed down during the entire course of the experiment as per the restraint protocol described above.

### Endurance

Climbing endurance was assessed in groups of at least 160 flies^30^ with the following modifications. Briefly, 8 vials of 20 flies from each cohort were subjected to the fatigue assay at two different time points: once on day 5 (to ensure no baseline differences unrelated to CI) and once on day 25 after the completion of exercise training. For each assessment, the flies were placed on the Power Tower exercise machine^37^ and allowed to climb until they were fatigued. Monitored at 15 min intervals, a vial of flies was visually determined to be “fatigued” when 10% or fewer flies could climb higher than 1 cm after four consecutive drops. Each vial was plotted as a single datum. Endurance experiments were conducted in three completely separate repetitions and scored blindly by separate experimenters. In all three repetitions, the rank was identical but the magnitude of the relative differences between groups varied by day and experimenter. All three repetitions were plotted as subcolumns to generate a single bar graph in GraphPad Prism. Mean time to fatigue was analyzed using one-way ANOVA with Tukey’s multiple comparisons test, also in GraphPad Prism (San Diego, CA, USA). All fatigue experiments discussed in the manuscript are represented in the main figures.

### Climbing speed

Climbing speed was assessed in Rapid Integrative Negative Geotaxis (RING) assays^62^ using groups of 100 flies per cohort with some modifications^58,63^. Five vials containing 20 flies each were set up in a RING apparatus and negative geotaxis stimulus was initiated by tapping the apparatus down on the countertop twice firmly and rapidly. The height of the flies in the vials was captured by photo image 2 s after the start of climbing. Flies were longitudinally tested twice per week to assess climbing ability at each aged time point. Raw image files obtained for each cohort were analyzed by semi-automated methods using ImageJ software (ImageJ version 1.53a, http://imagej.nih.gov/ij) to eliminate examiner bias. Briefly, a single raw image was used to develop a macro code with parameters able to binarize and assign a pixel height value for each individual fly pictured in the image. These same macro parameters were then applied to batch process all images in the data set under the exact same conditions and to generate the average values. Climbing speed was assessed blindly whenever possible in at least 3 repetitions by 2 different labs. To account for slight variations in working distance between experimenters, negative geotaxis performed in the Wessells lab was converted to quadrants as in Piazza et al.^25^while experiments performed in the Sujkowski lab are presented as raw data^63^. All mobility experiments were analyzed by linear regression with statistical significance determined by differences in slope and y-intercept. All repetitions of mobility discussed in the paper are presented in either the Figures or Supplemental Figures.

### Lifespan

Flies were flipped into fresh food every other day and the number of deaths was recorded. Dead flies were removed and counted until no flies remained. Non-natural deaths like escape or accidental death were censored from the result and did not exceed 10% of flies for any group. Survivability was analyzed following censoring using log-rank analysis in GraphPad Prism (San Diego, CA). Lifespan assays were performed in triplicate with each individual graph depicting a representative biological repetition. All longevity experiments discussed in the paper are presented in either the Figures or Supplemental Figures.

### Western blotting

Western blotting was performed with homogenate from thoraxes of 10 flies per group. Flies were homogenized in 95 °C lysis buffer (50 mM Tris pH 6.8, 2% SDS, 10% glycerol, 100 mM dithiothreitol), sonicated over ice for 15 s, boiled at 98 °C for 10 min, and centrifuged at 13,300 x g at room temperature for 10 min. Samples were electrophoresed on 4–20% gradient gels (Bio-Rad). ChemiDoc (Bio-Rad) was used to image western blots, which were then quantified using ImageLab (Bio-Rad, Hercules, CA). The following primary antibodies were used: anti-phospho-AKT (Ser473) (9271, 1:1000, Cell Signaling Technology), anti-AKT (4691, 1:1000, Cell signaling technology), anti-Ubiquitin (P37, 58395, 1:1000, Cell signaling technology). Secondary antibody: peroxidase conjugated anti-Rabbit (1:5000, Jackson Immunoresearch). For direct blue staining, PVDF membranes were submerged for 10 min in 0.008% Direct Blue 71 (Sigma-Aldrich) in 40% ethanol and 10% acetic acid, rinsed in 40% ethanol/10% acetic acid, air dried, and imaged. Western blots were performed using 3 biological replicates and 2 technical replicates per genotype, and statistical analysis was performed in GraphPad Prism.

### Immunofluorescence and confocal microscopy

Indirect flight muscle dissection and staining were done as described^64^ with minor modification. Adult flies were anesthetized in light CO_2_ and thoraces were prepared by removing head, legs, and abdomen with scissors. Thoraces were then incubated in relaxing buffer (RB) for 15 min, fixing it in 4% paraformaldehyde (PFA) in RB for 30 min, washing twice for 10 min each in washing buffer (1X RB with 0.3% Triton X-100) and bisecting them sagittally with a sharp microtome blade. Hemi-thoraces were incubated in RB for 15 min, fixed in 4% PFA in RB for 15 min, washed three times for 10 min each in washing buffer. For immunohistochemistry, hemi-thoraces were prepared as above and blocked in blocking buffer (1X PBS with 2% bovine serum albumin and 0.3% Triton X-100) for 2 h. The following day, tissues were washed three times for 10 min each in washing buffer and incubated in phalloidin AF488 (1:500, ThermoFisher) for 2 h in blocking buffer. Samples were washed three times for 10 min each in washing buffer and mounted in 50% glycerol. All steps were performed at room temperature, unless otherwise stated.

Fluorescent images were analyzed using ImageJ (NIH, Bethesda, MD, USA) and Fiji (ImageJ)^65^. At least 5 adult flies were analyzed for each group and triplicate biological cohorts were assessed. Significance was determined by one-way ANOVA with Tukey’s post-hoc comparison using GraphPad Prism (San Diego, CA).

#### Statistical analysis

All graphing and statistical analyses were performed by GraphPad Prism (version 10.5.0, https://www.graphpad.com, San Diego, CA, USA.) All experiments are represented either in the primary manuscript or the supplemental information.

## Supplementary Information

Below is the link to the electronic supplementary material.

Supplementary Material 1

## Data Availability

Data is provided within the manuscript or supplementary information files.
