# Supplementary material for: A new Drosophila model of prolonged inactivity shortens lifespan and impairs muscle function
Source: Sci Rep. 2025 Jul 31;15:27908. doi: 10.1038/s41598-025-13446-w (PMC12314096; doi:10.1038/s41598-025-13446-w)
Supplement: Supplementary file 1 — Supplementary Material 1 [file 41598_2025_13446_MOESM1_ESM.pdf]

A new *Drosophila* model of prolonged inactivity shortens lifespan and impairs muscle function

Jodi Protasiewicz[1], Sarah Snider[1], Mousumee Khan[2,3], Li Tao[2], Robert J. Wessells[2], and Alyson Sujkowski[1,4*]

[1]Department of Pharmacology, Wayne State University School of Medicine, Detroit, MI 48201, USA

[2]Department of Physiology, Wayne State University School of Medicine, Detroit, MI 48201, USA

[3]Department of Ophthalmology, Visual, and Anatomical Sciences, Wayne State University School of Medicine, Detroit, MI 48201, USA

[4]Department of Physical Therapy, Wayne State University Eugene Applebaum College of Pharmacy and Health Sciences, Detroit, MI 48201, USA

***Corresponding author** Alyson Sujkowski: Wayne State University School of Medicine, Department of Pharmacology, Detroit, MI 48201, USA. Email: asujkows@med.wayne.edu

Supplemental Methods

Quantitative real-time PCR

Total RNA was extracted from 20 isolated thoraxes using Trizol (Invitrogen). One-step quantitative real-time PCR (qRT-PCR) was done using Power SYBR Green PCR master mix and conducted using an ABI 7300 Real Time PCR System (Applied Biosystems).

Primer sequences:

Srl F: CTCTTGGAGTCCGAGATCCGCAA

Srl F: GGGACCGCGAGCTGATGGTT

dSestrin F: ATGTACTACGCCGTCGATTACT

dSestrin R: TCGTCCATGTCAAAGTCGGAT

Idit F: AGCAACGACATCGAGCTGAA

Idit R: CGCTAGCAGACTAGTCACGG

Act5C F: CGCAGAGCAAGCGTGGTA

Act5C R: GTGCCACACGCAGCTCAT

For each experimental group, at least three independent biological replicates with three technical repetitions were performed. Differences between groups were analyzed by one-way ANOVA with Tukey’s post hoc comparison in GraphPad Prism (San Diego, CA, USA).

Supplemental Figures

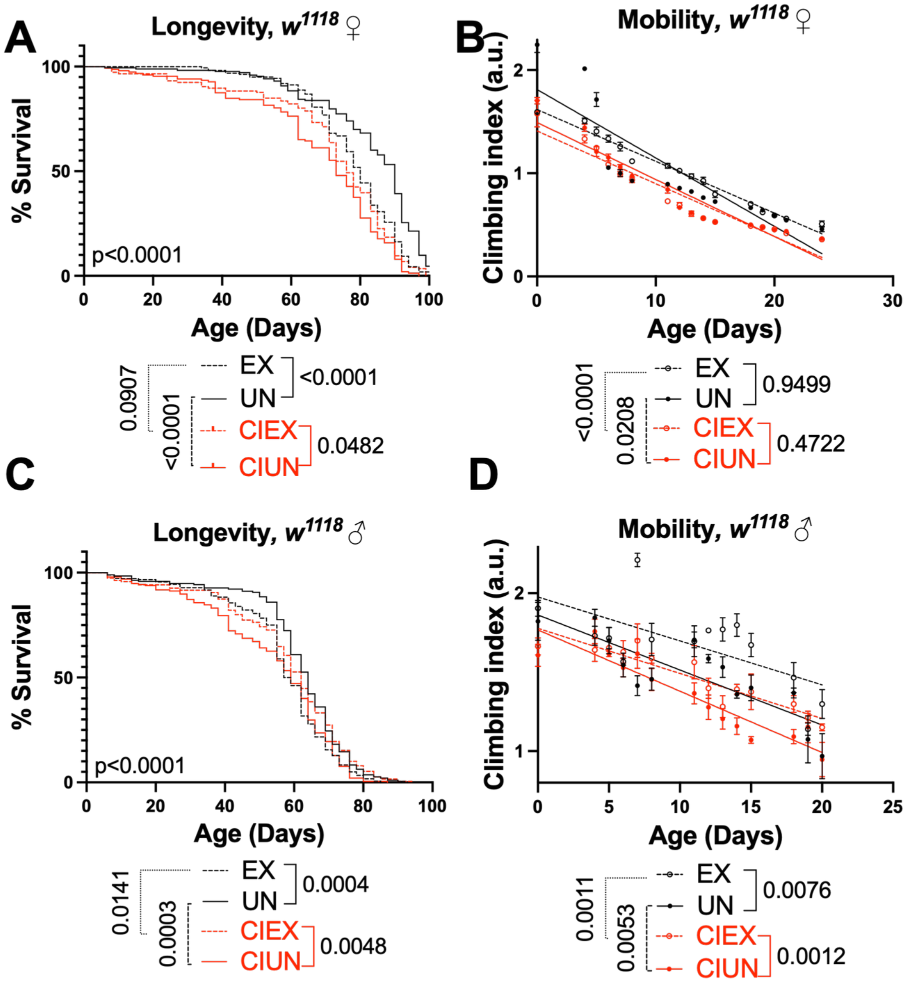

Supplemental Figure S1: Additional confirmation of restraint and exercise effects in w^{1118} flies. (A) Lifespan is improved in restrained female flies that complete 3 weeks of exercise when compared to unexercised, restrained siblings. **(B)** In this biological cohort, female flies that undergo CI have worse mobility than freely mobile flies whether exercised or not. A second cohort of exercised male flies that undergo mobility restriction have better lifespan **(C)** and mobility **(D)** than unexercised restrained siblings. n>200 flies per experiment. Exercise, mobility and longevity experiments were repeated a minimum of 3 times and in 3 genetic backgrounds (w^{1118}, y^1w^1, and Canton S). Trends were similar in all genotypes, with representative cohorts depicted here. w^{1118}, and y^1w^1 males showed the most consistent phenotypes in response to both restraint and exercise and were therefore used to complete the remaining experiments. Longevity analyzed by log-rank, mobility analyzed by linear regression, looking for differences in slope and intercept.

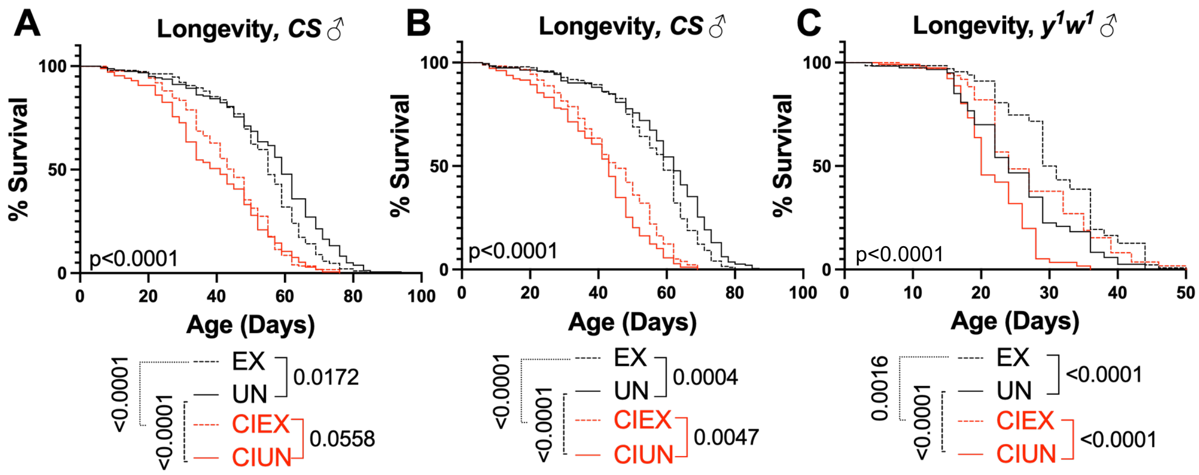

Supplemental Figure S2: Lifespan is improved in restrained exercised flies of multiple genetic backgrounds. Lifespan was assessed in duplicate biological replicates **(A, B)** of male *Canton S* flies under restraint stress. Exercise improved longevity in restrained flies but in one repetition **(A)** failed to achieve significance. **(C)** Exercise significantly improved lifespan in confined y^1w^1 males. Analyzed by log-rank

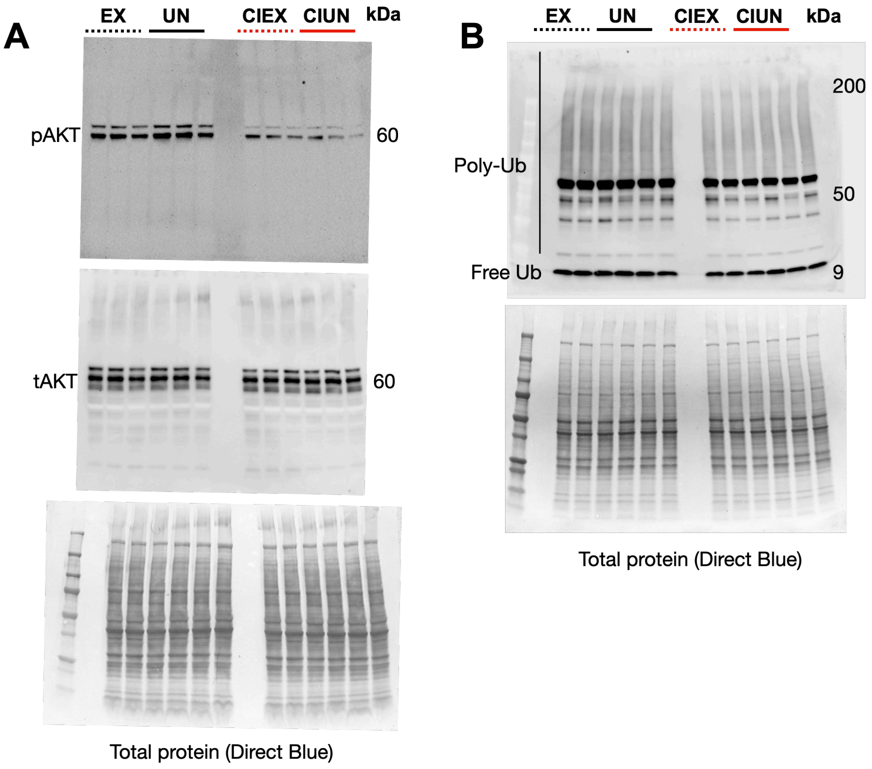

Supplemental Figure S3: Source data from Fig. 5; exercise preserves muscle structure in confined flies. Uncropped, unedited images from Figs. **(A)** 5D and **(B)** 5E.

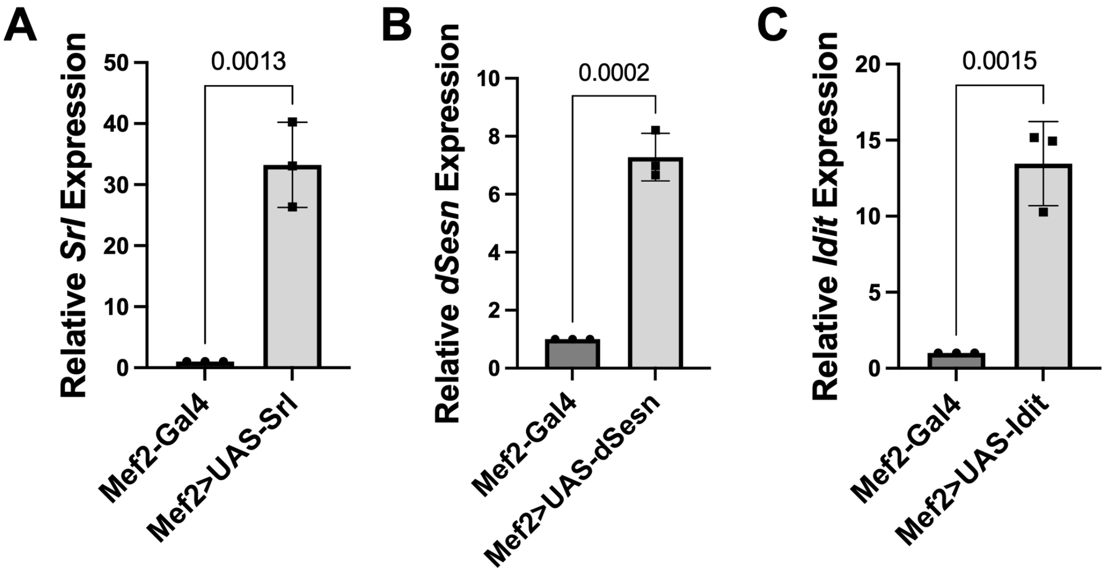

Supplemental Figure S4: Validation of muscle-specific overexpression in flies from Fig. 6. qRT-PCR for indicated genes in isolated thoraxes from flies with muscle-specific overexpression of **(A)** *Spargel*, **(B)** *dSesn*, or **(C)** *Idit*. Expression is relative to age-matched background control flies. 3 biological replicates of 20 thoraxes per replicate. Analyzed by student T-test.

Supplemental Legends

Supplemental Figure S1: Additional confirmation of restraint and exercise effects in *w^{1118}* flies. (A) Lifespan is improved in restrained female flies that complete 3 weeks of exercise when compared to unexercised, restrained siblings. **(B)** In this biological cohort, female flies that undergo CI have worse mobility than freely mobile flies whether exercised or not. A second cohort of exercised male flies that undergo mobility restriction have better lifespan **(C)** and mobility **(D)** than unexercised restrained siblings. n>200 flies per experiment. Exercise, mobility and longevity experiments were repeated a minimum of 3 times and in 3 genetic backgrounds (w^{1118}, y^1w^1, and Canton S). Trends were similar in all genotypes, with representative cohorts depicted here. w^{1118}, and y^1w^1 males showed the most consistent phenotypes in response to both restraint and exercise and were therefore used to complete the remaining experiments. Longevity analyzed by log-rank, mobility analyzed by linear regression, looking for differences in slope and intercept.

Supplemental Figure S2: Lifespan is improved in restrained exercised flies of multiple genetic backgrounds. Lifespan was assessed in duplicate biological replicates **(A, B)** of male *Canton S* flies under restraint stress. Exercise improved longevity in restrained flies but in one repetition **(A)** failed to achieve significance. **(C)** Exercise significantly improved lifespan in confined y^1w^1 males.

Supplemental Figure S3: Source data from Fig. 5; exercise preserves muscle structure in confined flies. Uncropped, unedited images from Figs. **(A)** 5D and **(B)** 5E.

Supplemental Figure S4: Validation of muscle-specific overexpression in flies from Fig. 6. qRT-PCR for indicated genes in isolated thoraxes from flies with muscle-specific overexpression of **(A)** *Spargel*, **(B)** *dSesn*, or **(C)** *Idit*. Expression is relative to age-matched background control flies. 3 biological replicates of 20 thoraxes per replicate. Analyzed by student T-test.
